# Identifying and overcoming COVID-19 vaccination impediments using Bayesian data mining techniques

**DOI:** 10.1038/s41598-024-58902-1

**Published:** 2024-04-13

**Authors:** Bowen Lei, Arvind Mahajan, Bani Mallick

**Affiliations:** 1 Department of Statistics, Texas A&M University, College Station, TX USA; 2 Department of Finance, Texas A&M University, College Station, TX USA

**Keywords:** Health care economics, Health policy, Data mining, Statistical methods

## Abstract

The COVID-19 pandemic has profoundly reshaped human life. The development of COVID-19 vaccines has offered a semblance of normalcy. However, obstacles to vaccination have led to substantial loss of life and economic burdens. In this study, we analyze data from a prominent health insurance provider in the United States to uncover the underlying reasons behind the inability, refusal, or hesitancy to receive vaccinations. Our research proposes a methodology for pinpointing affected population groups and suggests strategies to mitigate vaccination barriers and hesitations. Furthermore, we estimate potential cost savings resulting from the implementation of these strategies. To achieve our objectives, we employed Bayesian data mining methods to streamline data dimensions and identify significant variables (features) influencing vaccination decisions. Comparative analysis reveals that the Bayesian method outperforms cutting-edge alternatives, demonstrating superior performance.

## Introduction

The emergence of COVID-19 has greatly impacted people’s lives since 2020 and will continue to do so. The Center for Systems Science and Engineering (CSSE) at Johns Hopkins University^1^ reports that there have been more than 676 million cases and 6.8 million deaths in the world. To combat COVID-19, there are a number of restrictive methods to inhibit the spread of the virus^2–5^. These include lockdowns, quarantine, etc. These methods are widely used in many countries but many studies raise concerns about the costs and side effects of their use^6–10^, such as loss of gross domestic product (GDP), educational opportunities, increased deaths, higher mental health risks, and other societal costs. In addition to these restrictive methods, vaccines are another potent way to tackle the pandemic^3,11,12^. Higher vaccination rates would bring many benefits. However, the facts show that many people are unable or hesitant to get vaccinated^12–19^. In our study, impediments to COVID-19 vaccination are defined as unwillingness or refusal to receive the COVID-19 vaccine, or inability to receive the COVID-19 vaccine due to lack of vaccine availability ( CDC provides a definition of vaccination hesitancy measure at the following link https://data.cdc.gov/stories/s/Vaccine-Hesitancy-for-COVID-19/cnd2-a6zw/. However, this is a subset of our impediment measure since CDC hesitancy measure doesn’t consider the lack of availability.) We aim to predict vaccine impediment using Bayesian technique and to identify groups of important variables that contribute to impediments to vaccination. We then make policy recommendations to address impediments to vaccination. The World Health Organization (WHO) has recognized vaccine impediment as one of the top ten global health threats, as it can lead to low vaccination rates and the resurgence of preventable diseases. This impediment can stem from a variety of reasons.

**Figure 1:**
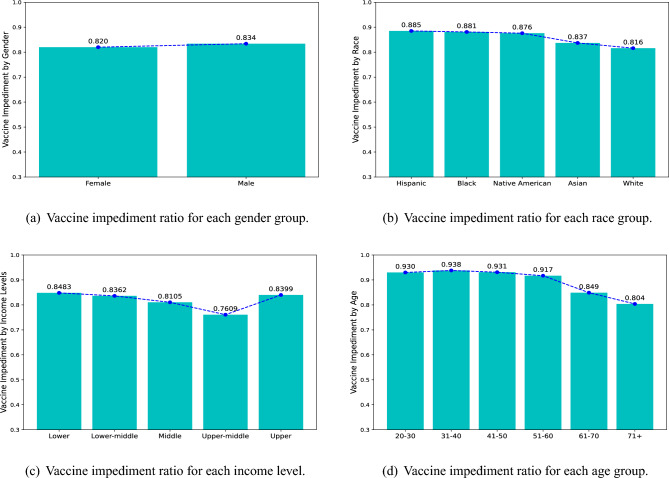
Vaccine impediment grouped by variables (**a**) gender, (**b**) race, (**c**) income level, and (**d**) age.

In this paper, we conducted an analysis of data sourced from a prominent health insurance provider in the United States. We briefly present how the vaccine impediment varies across insured populations, including gender, race, income level, and age, as shown in Fig. 1. In terms of gender, similar to results of previous research^20^, we can see that women and men have almost the same percentage of vaccine impediments, with males having slightly higher impediments to vaccination. For different racial groups, Whites and Asians get relatively low impediment scores, while Hispanics, Blacks, and Native Americans have higher scores based on the data. A similar pattern has been found in existing works^21^. For each income level, people tend to be more willing to vaccinate as their income increases, from the lower to the upper middle class, which is also found in other studies^22^. However, the upper class is similar to the lower class, who are hampered in terms of vaccination. For different age groups, young and middle-aged people (from 20 to 50 years old) have very similar rates and are more hesitant to be vaccinated. In contrast, older people are more willing to vaccinate and the willingness increases with age (from 51 years and older). This is consistent with the findings of existing studies^22–24^.

Impediments to vaccination are influenced by a variety of factors, and our goal was to gain deeper insights into the obstacles preventing individuals from getting vaccinated, identify them at an early stage, and formulate data-driven policies to address these challenges. This paper makes significant contributions to the existing literature in two key aspects. Firstly, it leverages granular and objective data obtained from a major health insurance provider, enabling a more in-depth and comprehensive analysis. Secondly, we employ an advanced classification model to predict the likelihood of a member being hesitant to receive the vaccine, yielding more accurate results compared to other statistical methods. Although we have used COVID-19 vaccination data, most of the results will likely be applicable to other epidemic or pandemic vaccination situations.

In this study, we introduce a two-stage methodology. In the first stage, we employ Bayes factor^25,26^ for preliminary screening, followed by the application of a Bayesian nonparametric regression technique known as Bayesian Multivariate Adaptive Regression Splines (BMARS)^27–29^ in the second stage. This approach is applied to population characteristic data provided by a major health insurance provider, with the aim of identifying barriers to vaccination. The pre-screening step enables our approach to effectively handle high-dimensional feature spaces by selecting the key features, simplifying the complex problem within the Bayesian framework. Additionally, the BMARS regression method allows for the modeling of nonlinear relationships between these selected key features and the response variable.

In the following sections, we first describe our Bayes-factor-based pre-screening and BMARS-based classification modeling (B-BMARS) method and introduce the vaccine impediment dataset to identify vaccination impediments. We then compare the results of B-BMARS with other popular baseline methods and analyze which variables play a key role in impeding getting vaccinated. Next, based on the modeling results, we present analyses and policy implications from the business perspective. We also describe other alternative baseline forecasting methods in the Supplementary Information.

## Methods

We propose a novel two-stage method to accurately and efficiently analyze people’s impediments to receiving the COVID-19 vaccine with proper selections of interpretable variables and their interactions. The first stage, pre-screening, is based on the Bayes factor, a widely used Bayesian method to quickly check the correlation between variables and response. Thus, we can effectively filter out apparently irrelevant variables and avoid unnecessary computational burdens and modeling challenges. In the second stage of BMARS-based classification, the unknown function is fitted by product-based spline basis functions, which can automatically fine-tune the selection of key variables and their interactions.

### Stage I: Bayes-factor-based pre-screening

In our COVID-19 vaccination data analysis, the dimension of potential key variables is usually too high to use Bayesian nonparametric models directly. Therefore, it is necessary to reduce the dimensionality of the variable space. We propose to take advantage of the model comparison ability of the Bayes factor and use it as a screening step to reduce the dimensions. Since our goal is to predict vaccine impediments, it becomes a binary classification problem. Therefore, we chose a method widely used for classification tasks, the Probit model, in which the conditional probability of one of the two possible attitudes toward the vaccine is equal to a linear combination of the underlying variables, transformed by the cumulative distribution function of the standard Gaussian^30,31^. For classification tasks, a widely used approach is to combine the regression model with a probit model using auxiliary variables. Specifically, in the classification framework, we use *z* to denote the observed response, which is a binary variable and *y* as the auxiliary variable. We assume the binary *z* to be 1 if $y>0$ and 0 otherwise. For the probabilistic model, it is defined as $p(z=1|y)=\Phi (y)$ where $\Phi$ is the standard Gaussian cumulative distribution function and *y* is defined as $y \sim \mathcal {N}(\varvec{ \beta }{\textbf {x}}+\beta _0, \sigma ^2)$ where $\varvec{ x}$ is the $p^*$ dimensional explanatory variables (covariates), $\varvec{ \beta }$ is the vector of regression parameters and $\sigma ^2$ is the error variance.

High-dimensional data analysis is always a daunting task. When the dimension $p^*$ is high, we run into a problem called “the curse of dimensionality”^32^. Though the high dimensional variables usually provide more information, they also lead to higher computational costs. The convergence of optimization algorithms or Bayesian sampling in a space of high dimensions is usually very slow. Also, it can harm the estimation accuracy, which is due to the difficult search in a space of high dimensions. Therefore, an effective and accurate variable selection is essential in high-dimensional modeling.

Pre-screening is a popular way to quickly filter out unimportant variables, making variable selection more efficient in a much lower-dimension space using a simpler model (like linear model), especially for ultrahigh-dimensional cases. In pre-screening methods, it is usually assumed that if one variable is important when predicting the response, it will be marginally associated with the response. Different measurements of the association are studied using, for example, p-value^32–34^. However, the pre-screening technique have not been fully explored in the Bayesian paradigm.

We use an off-the-shelf Bayesian method, Bayes factor^35,36^, for pre-screening. More specifically, the Bayes factor is a Bayesian alternative to classical hypothesis testing, which plays an important role in the model comparison and selection process. Essentially, the Bayes factor serves as a measure of how strongly data support a specific model compared to another. The Bayes factor is defined as a ratio of the marginal likelihood of two candidate models, typically regarded as a null and an alternative hypothesis. The general formula is as below.

$$\begin{aligned} \text {Bayes}\ \text {factor} = \frac{p(D|M_1)}{p(D|M_2)} = \frac{p(M_1|D)p(M_2)}{p(M_2|D)p(M_1)} \end{aligned}$$

where *D* denotes the available data and $M_1$ and $M_2$ denote two potential models. A larger value of this ratio indicates more support for $M_1$, and vice versa.

More specifically, to check the effect of the *j*th variable $x_{j}$ with the corresponding regression parameter $\beta _{j}$, we calculate the Bayes factor ($\text {BF}_j$) via Probit regression model as below

$$\begin{aligned} \text {BF}_j = \frac{p({\textbf {z}} | \mathcal {H}_1)}{p({\textbf {z}} | \mathcal {H}_0)}, \end{aligned}$$

where hypothesis $\mathscr {H}_1$ assumes that $y \sim \mathcal {N}(\beta _j x_j+\beta _0, \sigma _{j}^2)$, hypothesis $\mathscr {H}_0$ assumes that $y \sim \mathcal {N}(\beta _0, \sigma ^2)$, prior for $\beta _j$ is Gaussian distribution $p(\beta _j)\sim \mathcal {N}(0,\alpha )$, and use conjugate prior for the variances.

To compute the intractable marginal likelihood $p({\textbf {z}} | \mathscr {H}_1)$ (integrated over $\varvec{ \beta }$), we choose to use Laplace Approximation^37–39^. Specifically, under $\mathscr {H}_1$, the posterior distribution of $\beta _j$ is

1 $$\begin{aligned} p(\beta _j | D)&\propto p(D | \beta _j) p(\beta _j) = f(\beta _j), \end{aligned}$$

2 $$\begin{aligned} \log f(\beta _j)&= \log p(D | \beta _j) | \log p(\beta _j) = \sum _{i=1}^N \log \Phi (z_i\beta _j x_{ij}) - \frac{1}{2}\beta _j^2. \end{aligned}$$

Suppose $\beta _j^*$ is a maximum of *f*, we can calculate the negative Hessian at $\beta _j^*$

3 $$\begin{aligned} A = - \nabla \nabla \log f(\beta _j^*) = \sum _{i=1}^N [v_i(s_i + v_i)x_{ij}^2] + 1, \quad v_i = \frac{\mathcal {N}(s_i | 0, 1)}{\Phi (s_i)}, \quad s_i = z_i\beta _j x_{ij}. \end{aligned}$$

Then, the approximate posterior can be written as $Q(\beta _j) = \mathcal {N}(\beta _j | \beta _j^*, A^{-1})$. Thus, we can approximate the marginal likelihood

4 $$\begin{aligned} p(D | \mathscr {H}_1) \approx \prod _{i=1}^N \int p(\varvec{z} | \beta _j) Q(\beta _j) d\beta _j = \prod _{i=1}^N \Phi \left (\frac{z_i\beta _j x_{ij}}{\sqrt{x_{ij}A^{-1}x_{ij} + 1}}\right ). \end{aligned}$$

A larger value of $\text {BF}_j$ suggests our preference for the hypothesis $\mathscr {H}_1$ to the hypothesis $\mathscr {H}_0$, implying a potential key role of ${\textbf {x}}_j$ when predicting ${\textbf {z}}$. Then after calculating $\{\text {BF}_j, j=1,\cdots ,p\}$, we can choose the top ranked variables with respect to $\text {BF}_j$. Say we select *p* explantory variables out of $p^*$ variables. Next, we use these *p* selected variables $\varvec{ x}$ for the Bayesian nonparametric classification model.

### Stage II: BMARS-based classification modeling

In stage 2, we use a flexible nonlinear method to relate the response *z* with the selected explanatory variables from step 1. More specifically, we use Bayesian multivariate adaptive regression splines (BMARS)^27,28^ which is a Bayesian version of a flexible non-parametric regression and classification method named MARS^40^. We extend the previously defined linear probit model for nonlinear modeling using product spline basis functions. We use the probit model defined in the previous section, for the *i*th observation $p(z_{i}=1|y_{i})=\Phi (y_{i}), (i=1,\cdots ,n)$. Next we use BMARS to relate the auxilary variables *y* with the explanatory variables ${\textbf {x}}$ through a regression model. In BMARS, for regression tasks, the product-based spline basis functions are not only used to model the unknown function *f*, but also automatically select the nonlinear interactions among the variables. The mapping function between the selected variables ${\textbf {x}}_i \in \mathscr {R}^p$ and the auxiliary variable $y_i$ as below

5 $$\begin{aligned} y_i&= f({\textbf {x}}_i) + \varepsilon _i, \quad \hat{f}({\textbf {x}}_i) = \sum _{j=1}^m \alpha _j B_j({\textbf {x}}_i), \quad \varepsilon _i {\mathop {\sim }\limits ^{\text {i.i.d}}} \mathcal {N}(0, \sigma ^2), \end{aligned}$$

where *m* is the number of basis functions and $\alpha _j$ denotes the coefficient for the basic function $B_j$ which is designed as

6 $$\begin{aligned} B_j({\textbf {x}}_i) = \left\{ \begin{array}{rcl} &{}1, &{}j=1 ,\\ &{}\prod _{q=1}^{Q_j} [s_{qj}\cdot ({\textbf {x}}_{i,v(q,j)} - t_{qj})]_+, &{}j\in \{2,3,\cdots ,m\} \end{array} \right. \end{aligned}$$

where the $s_{qj} \in \{-1,1\}$, the *v*(*q*, *j*) denotes the index of the variables and the set $\{v(q,j);q=1,\cdots ,Q_j\}$ are not repeated, the $t_{qj}$ refers to the partition location, $(\cdot )_+ = \max (0,\cdot )$, and $Q_j$ is the polynomial degree of the basic function $B_j$ and also indicates the number of variables involved in $B_j$.

For probit model, the posterior distribution is not available in explicit form so we use Markov Chain Monte Carlo (MCMC) algorithm to simulate from the posterior distribution. As the dimension of the model *m* is unknown, we use the reversible jump Metropolis-Hastings algorithm^41^. More specifically, the model parameters we are interested in within the Bayesian framework of BMARS^27^ are assumed to include the number of basis functions *m*, as well as their degree of interaction $Q_j$, their coefficients $\alpha _j$, their associated split points $t_{qj}$, and the sign indicators $s_{qj}$. We can use $\varvec{\theta }^{(m)} = \{ \mathscr {B}_1,\cdots ,\mathscr {B}_m \}$ where $\mathscr {B}_j$ to denote the model parameters $(Q_j, \alpha _j, t_{1j}, \cdots , t_{Q_j,j}, s_{1j}, \cdots , s_{Q_j,j})$ for each basis function $B_j$. Then, the hierarchical model can be written as

7 $$\begin{aligned} p(m, \varvec{\theta }^{(m)}, {\textbf {y}}) = p(m)p(\varvec{\theta }^{(m)}|m)p({\textbf {y}}|m, \varvec{\theta }^{(m)}), \end{aligned}$$

and the joint posterior for parameters *m* and $\varvec{\theta }^{(m)}$ can be written in the following factorized form

8 $$\begin{aligned} p(m, \varvec{\theta }^{(m)} | {\textbf {y}}) = p(m|{\textbf {y}}) p(\varvec{\theta }^{(m)} | m,{\textbf {y}}). \end{aligned}$$

In this algorithm, we update the model randomly using one of three steps, including (a) changing a node position, (b) creating a basis function, or (c) deleting a basis function, and then correcting the proposed new sample by the Metropolis-Hastings step^42,43^. Under this sampling scheme, samples based on significant variables are more likely to be accepted, which enables automatic feature selection by the algorithm and is important for us to make policy implications.

## Data description

To understand vaccine impediments, we analyze a dataset obtained from one of the major health insurance providers in the United States. Since the dataset comes from the insured population, our analysis of impediments to vaccination and potential policy implications focuses on the insured population. More specifically, the dataset includes a total of 974,842 observations, each presenting information about one member of the insurance provider, with 1 binary response and 368 variables. About 69% of the variables are numeric and the remaining 31% are categorical. We note that we use synthetic data based on real data, which maintains all relationships within the dataset but is not specific to any individual insured person. This minimizes the risks associated with privacy to share protected data.

### Response measures

The data records whether an insurance member is vaccinated or not. We assume that if a member is not vaccinated, then that member has some sort of impediment to vaccination. We use a broad definition of impediments that includes various reasons such as not believing in the efficacy of the vaccine, barriers like lack of resources, inability, or ideological/political reasons, etc.

### Variables

The data document a number of characteristics of insured members that are potential variables influencing their willingness and availability to receive vaccines. These variables can be categorized into eight groups of characteristics, including medical claims, pharmacy claims, laboratory claims, demographics, credit data, condition-related data, centers for Medicare & Medicaid services (CMS) features (original reasons for entry into Medicare), and other characteristics. In total, there are 253 numerical variables and 115 categorical variables. The detailed descriptions of each group are provided in Table 1.

**Table 1:** Variable group description. Potential variables for insured members are defined as those that can influence a member’s willingness and availability to be vaccinated, which can be categorized into eight groups of characteristics.

Feature group	Description
Medical claims	This category of data includes utilization by categories, such as inpatient, emergency, and outpatient, to name a few. In addition, authorization and costs by condition, as well as inpatient claims data, can be found here.
Pharmacy claims	This category of data includes costs of prescriptions, brands covered, generic prescriptions, mailed or non-mailed prescriptions, maintenance prescriptions, generic product identifier (GPI) level prescription usage, etc.
Laboratory claims	This category of data includes abnormal laboratory outcome indicators, as well as more subdivided abnormal laboratory outcome indicators by category such as cholesterol, estimated glomerular filtration Rate (eGFR), Hemoglobin, etc.
Demographics	This category of data includes gender, race, age, geography, census, income level, education level, household composition, homeowner status, etc.
Credit data of insured members	This category of data includes the percentage of all mortgage account balances in bankcard accounts (accounts that are severely depreciated), the number of all mortgage accounts (120 days past due or in repossession), and the percentage of high mortgage balances, etc.
Condition-related data	This category of data includes the count of claims by Charlson Comorbidity Index, CMS Diagnosis Code Categories, the percentage of claims associated with multiple chronic conditions (MCC), Diagnosis Code Categories, etc.
CMS features	This category of data includes disability, CMS risk score, CMS total payment amount, etc.
Other features	This category of data includes home health discharge, healthcare effectiveness data and information set (HEDIS) features, out-of-network provider costs, revenue code features, behavioral segmentation, etc.

### Data pre-processing

Before modeling the data, we use some pre-processing steps to make the data structure compatible with the model. First, for convenience, we transform each categorical variable into several dummy variables. Thus, when the data are put into the model, there are 898 variables. Second, to fairly compare the different models, we balance the two types of samples by using a sample of 10,000 vaccinated clients and a sample of 10,000 unvaccinated clients as training data. Similarly, we sample a balanced test data including 2,000 clients.

## Results

### Classification analysis: accuracy

In this section, in terms of accuracy, we compare our two-stage method (B-BMARS) with several widely-used classification models, including extreme gradient boosting (XGBoost)^44^, Gaussian process classification (GP)^45^, random forest (RF)^46^, and multilayer-perceptrons-based deep neural network (DNN)^47^, which have all demonstrated good performance in various applications. We use 0.5 as the threshold to calculate the accuracy, which is widely used for binary classification. For the overall analysis with different thresholds, we further use the area under curve (AUC) values^48^ for comparison (shown in the next Section). Specifically for our B-BMARS, in the first stage, we use Bayes factor to quickly examine the potential predictive power of each variable on the response. Then, in the second stage, we use B-BMARS to fit the unknown function between the key variables and the responses in a more refined manner. A detailed description can be found in the “Methods” section.

In the first stage of pre-screening, we experiment by keeping different numbers of the top variables where the pre-screening dimension $p_{scr} =50$ which is a proper value found empirically. We also compare the scenario without using pre-screening which corresponds to using all the 898 variables. However, using all the 898 variables is not practical with limited computational resources. Table 2 shows the accuracy among pre-screening dimension $p_{scr}=50$ and without the pre-screening step. Our proposed B-BMARS gives the highest accuracy 0.614 and beats other popular baseline alternatives. Random Forest’s best result is close to our B-BMARS result but always below it.

**Table 2:** Accuracy comparison with baseline methods under pre-screening dimension$p_{scr}=50$and without pre-screening step. We compare our B-BMARS with extreme gradient boosting (XGBoost), Gaussian process classification (GP), random forest (RF), and multilayer-perceptrons-based deep neural network (DNN). Our B-BMARS generally improves or maintains accuracy compared to other baseline methods.

Model	B-BMARS	XGBoost	GP	RF	DNN
$p_{scr}=50$	**0.614**	0.586	0.561	0.593	0.531
No Pre-screen	0.605	0.602	0.530	**0.612**	0.552

The largest value in each scenario is in bold.

**Figure 2:**
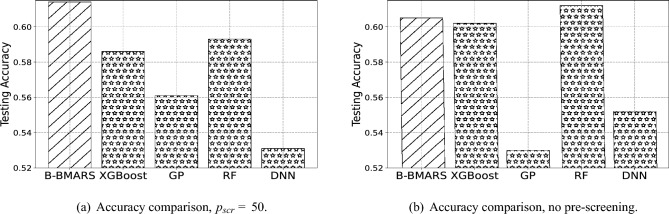
Visualization of accuracy comparison with baseline methods under pre-screening dimension $p_{scr}=50$ and without pre-screening step. We compare our B-BMARS with extreme gradient boosting (XGBoost), Gaussian process classification (GP), random forest (RF), and multilayer-perceptrons-based deep neural network (DNN). Our B-BMARS generally improves or maintains accuracy compared to other baseline methods.

We also visualize accuracy comparisons under pre-screening $p_{scr}=50$ and scenario without pre-screening step in Fig. 2a and b. The slash bars represent our B-BMARS, and the star bars represent XGBoost, GP, RF, and DNN from left to right, respectively. As we can see, the green bars are the highest in Fig. 2a, and also comparable to the highest blue columns in Fig. 2b. This indicates that our B-BMARS can maintain the performance for different scenarios. However, other baselines are relatively more sensitive to different settings. Additionally, we can see that RF achieves the best performance when $p_{scr}=898$, which leads to a high computational burden and is not practical with limited computational resources.

### Classification analysis using AUC values

Apart from the accuracy, we also choose AUC values^49^ to measure model performance, where higher AUC values indicate a better classifier. The baselines considered are aligned with the previous accuracy comparison. Table 3 shows the best AUC value among different pre-screening dimensions of each model. Our proposed B-BMARS gives the highest AUC value 0.651, followed by RF.

**Table 3:** The AUC value comparison with baseline methods under pre-screening dimension$p_{scr}=50$and without pre-screening step. We compare our B-BMARS with extreme gradient boosting (XGBoost), Gaussian process classification (GP), random forest (RF), and multilayer-perceptrons-based deep neural network (DNN). Our B-BMARS generally improves or maintains AUC value compared to other baseline methods.

Model	B-BMARS	XGBoost	GP	RF	DNN
$p_{scr}=50$	**0.651**	0.633	0.625	0.630	0.546
No Pre-screen	0.643	0.642	0.501	**0.648**	0.611

The largest value in each scenario is in bold.

**Figure 3:**
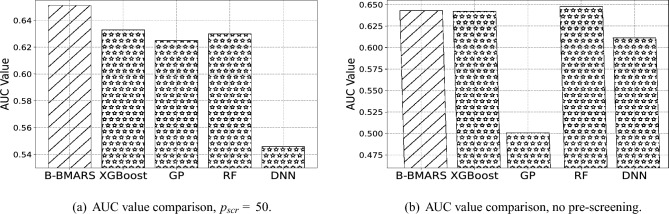
Visualization of the AUC value comparison with baseline methods under pre-screening dimension $p_{scr}=50$ and without pre-screening step. We compare our B-BMARS with extreme gradient boosting (XGBoost), Gaussian process classification (GP), random forest (RF), and multilayer-perceptrons-based deep neural network (DNN). Our B-BMARS generally improves or maintains AUC value compared to other baseline methods.

Similar to accuracy comparison, we also show detailed AUC value comparisons under pre-screening $p_{scr}=50$ and scenario without pre-screening step in Fig. 3a and b. The slash bars represent our B-BMARS, and the star bars represent XGBoost, GP, RF, and DNN from left to right, respectively. As shown in the figures, the green bars are the highest in Fig. 2a, and also comparable to the highest blue columns in Fig. 2b. This indicates that the classification rule from our B-BMARS is consistently one of the best classification rules in different pre-screening dimensions $p_{scr}$. However, other popular baselines have more fluctuations in different scenarios, with a drop in AUC values when resources are limited and pre-screening has to be used.

### Variable selection

B-BMARS is effective in selecting the most important variables. We find that there are four main categories of variables playing a key role in influencing the vaccine impediments of insured members, i.e., low household assets, high health risks, highly uninsured areas, and physician-related information. As shown in Table 4, we list ten interesting and important variables selected by our B-BMARS, along with their detailed descriptions and the categories to which they belong.

When trying to determine people’s willingness or ability to take the COVID-19 vaccine, it is helpful to look at their household asset status, and we find that people with low household assets will be hesitant to receive the vaccine, which is in line with existing research findings^50^. For example, among the significant variables listed, Supplemental Nutrition Assistance Program (SNAP) benefits per capita is selected^20^, which reflects whether people generally have a stable source of food and thus reflects their household asset status. It is also important to check the number of non-mortgage accounts that are more than 60 days past due^51^. If many non-mortgage accounts are chronically past due, it is likely that household assets are low. In addition, we can see that per capita income in Table 4 in the last 12 months is one of the key variables, which gives a direct indication of people’s economic situation.

Health risk is another important variable of COVID-19 vaccination propensity prediction, and people are more reluctant to get vaccinated if they already have a high health risk^52,53^. For example, the trend in the number of prescriptions per month is noteworthy. It represents a change in people’s health status and can indicate whether they are at high health risk. In addition, we need to look at the number of monthly prescriptions related to heart disease-heart failure medications, which also shows how often people are taking their medications and revealing their health status.

In addition to the categories mentioned above, the availability of better healthcare coverage in the area also affects people’s proclivity to get vaccinated, and populations living in highly uninsured areas are more unlikely to receive COVID-19 vaccination^54^. As listed in the key variables, the net monthly payments for behavioral health claims related to skilled nursing inpatient facilities have a significant impact. Also, trends in monthly prescription costs associated with vaccine drugs reflect health care coverage and indicate people’s attitudes to vaccinations, and the percentage of adults under age 65 without health insurance in the corresponding area is selected. The higher the percentage, the worse the health care coverage is.

Last but not least, there is a need to consider whether individuals trust their physicians and the public health system^55,56^; if individuals are not willing to use their physicians as their primary source of medical information, they are unlikely to be vaccinated^57^. For instance, we can get some information about people’s beliefs from the percentage of physician evaluations and claims management related to outpatient visits in the past year. A relatively high percentage score means that people are more likely to use their public health system and trust their doctors.

**Table 4:** The important variables selected by B-BMARS from potential variables for insured members of a major health insurance provider explaining vaccination impediment with the pre-screening dimension $p_{scr} = 50$.

Variable	Description	Category
Prescription Number Trend	Trend of the number of prescriptions per month in the past three months versus the third to sixth month prior to the score date.	High health risk
SNAP Benefits	Supplemental Nutrition Assistance Program (SNAP) benefits per capita.	Low household assets
Pass-due Non-mortgage Loan	The number of non-mortgage loan accounts that are more than 60 days past due.	Low household assets
Cardiology Prescription Number	Number of monthly prescriptions related to cardiology-heart failure drugs in the past year (based on insurance drug classification).	High health risk
Behavioral Health Payment	Net monthly payments for behavioral health claims related to skilled nursing inpatient facilities for the past ninth to twelfth month prior to the scoring date.	Highly uninsured areas
Generic Prescription Number Trend	Trend in the number of monthly prescriptions for generic drugs at the generic product identifier 6 (GPI6) level in the past third to sixth months compared to the sixth to ninth months prior to the rating date.	High health risk
Per Capita Income	Per capita income in the past 12 months 2014-2018.	Low household assets
Vaccine Prescription Cost Trend	Trends in monthly prescription costs associated with vaccine drugs in the past sixth through ninth months compared to the ninth through twelfth months prior to the score date (based on GPI2 subgroups).	Highly uninsured areas
Missing Insurance Percentage	Clinical Care - Percentage of adults under age 65 without health insurance.	Highly uninsured areas
Physician Evaluations and Claims	Percentage of physician evaluations and claims management related to outpatient visits in the past year.	Use of doctors’ info

### Non-linear basic function selection

**Figure 4:**
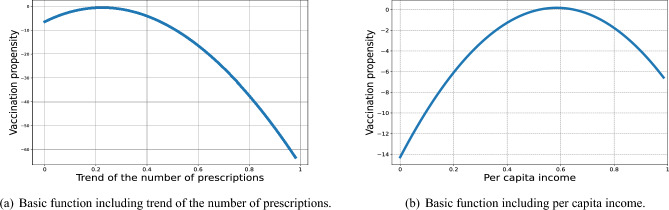
Curves of the non-linear basic functions selected by B-BMARS (**a**) trend of the number of prescriptions (Prescription Number Trend), and (**b**) per capita income (Per Capita Income).

To provide more interpretation of the selected variables, we plot a nonlinear basis function curve including the selected variables. We take two important variables, namely the trend of the number of prescriptions and per capita income, as examples. As depicted in Fig. 4a, the propensity to vaccinate begins to decline with increasing prescriptions when a small number of prescriptions is reached, indicating high health risks. In Fig. 4b, people first become more willing to vaccinate and then gradually become less willing to vaccinate as their per capita income increases, consistent with what we observed in our data overview.

### Interaction selection

B-BMARS is not only effective in selecting the most important variables but also in identifying variables that have significant interactions. As shown in Table 5, we list five important variable interactions selected by B-BMARS to predict COVID-19 vaccination propensity. Prescription count for cardiology is the most important and has many interactions with other variables^58^. Other variables are composed of health-related variables and financial conditions. Therefore, it is necessary to consider both the information related to the number of prescriptions and other important variables.

**Table 5:** The important variable interactions selected by B-BMARS from potential variables for insured members of a major health insurance provider explaining vaccination impediment with the pre-screening dimension $p_{scr} = 50$.

Variable 1	Description 1	Variable 2	Description 2
Cardiology Prescription Number	Number of monthly prescriptions related to cardiology-heart failure drugs in the past year (based on insurance drug classification).	Tier 1 Prescription Number	Number of prescriptions related to Tier 1 drugs per month in the three months prior to the score date.
Cardiology Prescription Number	Number of monthly prescriptions related to cardiology-heart failure drugs in the past year (based on insurance drug classification).	Prescription Number Trend	Trend in the number of prescriptions per month for the past three months compared to the number of prescriptions for the third through sixth months prior to the scoring date.
Cardiology Prescription Number	Number of monthly prescriptions related to cardiology-heart failure drugs in the past year (based on insurance drug classification).	Pass-due Non-mortgage Loan	The number of non-mortgage loan accounts that are more than 60 days past due.
Cardiology Prescription Number	Number of monthly prescriptions related to cardiology-heart failure drugs in the past year (based on insurance drug classification).	Generic Prescription Trend	Trend in monthly prescription costs associated with generic drugs over the past three months compared to the third through sixth months prior to the scoring date.
Cardiology Prescription Number	Number of monthly prescriptions related to cardiology-heart failure drugs in the past year (based on insurance drug classification).	Generic Prescription Number	Number of prescriptions related to anticonvulsant drugs per month (based on generic product identifier-2 grouping) for months six through nine prior to the scoring date.

## Business analysis and policy implication

Reducing vaccination impediments is important to slow the emergence of new virus variants. This will reduce the burden on patients and public health resources and will reduce costs incurred by insurance and healthcare providers. Thus, it is critical to develop targeted strategies to improve the ability to get vaccinated and reduce hesitancy. Vaccine impediment is a complex decision-making process influenced by a variety of contextual, individual and group, and vaccine-specific variables, including communication, socioeconomics, geographic barriers, vaccination experience, risk perception, and vaccination program design.

From our analysis in Section Variable Selection and Interaction Selection, it is clear that people with low assets, high health risks, low medical coverage, and distrust of doctors and the public health system are mostly reluctant to get vaccinated. Moreover, according to our analysis, these characteristics interact with each other. For example, people with low assets or low medical coverage have higher health risks. For these members, there are greater barriers than for other members. They may have fewer resources, more difficulty reaching vaccination sites, and less information about the nature of the pandemic.

We define the cause of COVID-19 vaccination impediments in these groups as due to physical barriers, psychological barriers, and health barriers. Physical barriers can be explained by having less access to the vaccine. Members with disabilities are likely to suffer from a lack of mobility. Psychological barriers can be explained by misunderstanding and mistrust. Members with few assets, low income, and high debt may live in communities where mistrust is prevalent or have fewer resources to obtain accurate information about the vaccine. Health barriers can be explained by high health risks, such as chronic diseases. The members may be older, living insecurely, or in poor health and worried about the side effects of vaccination.

### Physical barrier

**Potential policy implications to overcome challenges to access vaccination.** People tend not to get vaccinated if it is difficult and cumbersome to obtain the vaccination. Difficulties often arise from limited mobility due to disability or age, availability of time, transportation, and low supply of vaccinations.

- **Implication 1:** For people with limited mobility due to disability or age, we do not recommend that they visit a medical facility for vaccination, where there may be a high risk of cross-infection. We recommend that the health care provider provide home care services to help them get vaccinated at home.
- **Implication 2:** For people with limited time, lack of transportation, and selected constraints, access to vaccines is limited for these and other reasons like poor financial status. The health care provider can provide them with travel assistance, such as language instruction and transportation help. The provider can also arrange some special activities including vaccination camps near their homes to help them get vaccinated.
- **Implication 3:** For people in areas with low vaccine supply, it is more difficult for them to get vaccinated, even if they want to. Therefore, it is necessary to increase vaccine supply and reduce geographical inequalities. We recommend that the health care provider work with pharmacies to open more vaccination sites and productively send notifications to residents when vaccines are available.

### Psychological barrier

**Potential policy implications to overcome misunderstanding and mistrust of vaccine.** People tend not to get vaccinated if they have misconceptions about the vaccine and think it will be harmful to them. The source of these misconceptions can be family, friends, social media, or social norms. Or they ignore the need for vaccination because they are currently in good health.

- **Implication 1:** We recommend the health care provider get involved in community events and health activities to build stronger relationships with insured members. Then, it can select community leaders as vaccine ambassadors to deliver messages that allow vaccine recipients to share their reasons for vaccination, which will encourage people to reframe how they think about vaccines and build trust in the public health system.
- **Implication 2:** For people who do not understand the necessity of vaccination, they may not be motivated enough to get vaccinated because they are in good health. We recommend emphasizing the age-independent health benefits and importance of vaccination, providing them instructional videos or organizing lectures.

### Health barrier

**Potential policy implications to overcome high health risk.** People are more concerned about the side effects of vaccines if they are at high health risk. Specifically, they were concerned that the side effects of the vaccine would exacerbate their existing health problems.

- **Implication 1:** We recommend obtaining more information about their health to understand if the vaccine can negatively interact with their current medications and existing problems. They should be educated if the vaccine is indeed safe for them.
- **Implication 2:** We recommend using telemedicine to track their health after vaccination. This can prevent any unexpected health problems and make them feel more confident in the public health system.

### Expected benefit analysis

In this section, we use an example to analyze the expected benefits of utilizing our methodology and the resulting policy implications to address vaccine impediments. We have actual data from a major U.S. healthcare provider. It is a publicly listed company that is committed to maximizing benefits for its stakeholders, particularly its shareholders. Given the data available to us, and utilizing publicly available data from other sources, we would like to estimate the preventable costs and incremental costs associated with our proposal to determine the impact on the savings of the healthcare provider.

More specifically, we use an all-vaccination rate (VRate) of 19.55% as of March 31st, 2021, which is derived by dividing the number of U.S. all-vaccinated persons by the U.S. population. The number of U.S. all-vaccinated persons is 64,852,669 from the Centers for Disease Control and Prevention’s (CDC) COVID data tracker^59^, and the U.S. population is 331,791,631 according to the United States Census Bureau’s U.S. and World Population Clock^60^. In addition, we use a number of Medicare Advantage members of one of the major health insurance providers in the United States (N) of 4,600,000 in 2020. Therefore, we can approximate the number of impedimented members as $\text {N}_u$ = (1-VRate)$\cdot$N=3,700,700.

We then calculate the amount of preventable costs based on our B-BMARS method by encouraging more members to get vaccinated. From Centers for Medicare and Medicaid Services (CMS) report^61^, we obtain the average cost of medical services for COVID-19 hospitalizations and the number of medicare hospitalizations due to COVID-19 per 100,000 patients, which are $24,000 and 1,825, respectively. We also get the effectiveness of full vaccination in preventing hospitalization. According to the CDC’s August 2021 presentation in Morbidity and Mortality Weekly Report^62^, the effectiveness in adults 75 years or older is 91% for Pfizer-BioNTech, 96% for Moderna, and 85% for Janssen COVID-19 vaccines(CDC). Therefore, we choose 85% to approximate the lower bound of savings. Using our B-BMARS (as shown in Table 2), we are able to successfully identify 61.4% of impedimented members ($$\text {R}_s$$). Based on this information, we calculate the preventable costs for patients not vaccinated with COVID-19 via Equation (9). Specifically, the $$\text {Hospital}/100,000$$ calculates the percentage of people who receive Medicare hospitalizations. We multiply this by $$\text {R}_s$$ and $$\text {Ratio}$$ to represent the approximate proportion of people spared hospitalization by vaccination. We then multiply this by $$\text {N}_u$$ to get the approximate number of hospitalizations prevented by vaccination. Finally, we multiply this by $$\text {Fee}$$, which is the cost of patients preventable through vaccination. The results are shown in Table 6, and we successfully prevent more than 845 million dollars in costs.

9 $$\begin{aligned} \text {Prevent-Cost} = (\text {Hospital}/100,000) \cdot \text {R}_s \cdot \text {Ratio} \cdot \text {N}_u \cdot \text {Fee} \end{aligned}$$

**Table 6:** Summary of the average medicare fee for COVID-19 hospitalizations, the medicare COVID-19 hospitalizations per 100K persons, the successful identification rate of impedimented members, the effectiveness of full vaccination in preventing hospitalization, and the calculated preventable costs for patients not vaccinated with COVID-19. We can prevent 846 million dollars in costs by eliminating vaccine impediments.

Name	Abbreviation	Value
Average medicare fee-for-service COVID-19 hospitalizations	Fee	$24,000
Medicare COVID-19 hospitalizations per 100,000	Hospital	1,825
Successful identification rate of impedimented members	$$\text {R}_s$$	0.614
Effectiveness of full vaccination in preventing hospitalization	Ratio	0.850
Preventable costs for patients not receiving COVID-19 vaccine	Prevent-Cost	$845,951,155

In addition, we approximate the extra cost of the incremental vaccination. We collect relevant information from Centers for Medicare and Medicaid Services (CMS)^63^. Specifically, for those without disabilities, the cost of the vaccination is $80 per person, assuming 2 doses of each vaccine and a single dose cost to Medicare of $40. For those with disabilities, the cost of the home vaccination has increased to $150 per person because of an additional $35 per dose. In our dataset, the percentage of people with disabilities is 25%. Therefore, we can estimate the cost of having impedimented members vaccinated following Equation (10). Specifically, we multiply the ratios $$(1-\text {R}_d)$$ and $$\text {R}_d$$ by $$\text {N}_u$$ to give the approximate numbers of people without and with disabilities, respectively. Next, we multiply these two numbers by $$\text {R}_s$$ to get a rough estimate of the number of people in each impedimented group that we can successfully identify. This is then multiplied by the corresponding costs $$\text {Cost}_{nd}$$ and $$\text {Cost}_{d}$$, respectively. Finally, we add the estimated costs of the two groups to arrive at the final total extra cost. The result is $221,542,406 as shown in Table 7.

Based on all the above calculations, using Equation (11), we obtain a total savings of more than $**624 million** by addressing impediments to vaccination for the insured population. Specifically, we subtracted the total extra cost of vaccination from the total preventable costs due to vaccination to derive the total savings. We do not have a firm estimate of the marginal cost of implementing our policy recommendations. However, we are informed that it will be a small fraction of the $624 million savings calculated here. At a minimum, this number provides health insurance and healthcare providing organization guidance in developing a budget for implementing our policy recommendations. This example demonstrates how our methods can be transformed to a monetary value.

10 $$\begin{aligned}&\text {Extra-Cost} = (1-\text {R}_d) \cdot \text {R}_s \cdot \text {N}_u \cdot \text {Cost}_{nd} + \text {R}_d \cdot \text {R}_s \cdot \text {N}_u \cdot \text {Cost}_{d} \end{aligned}$$

11 $$\begin{aligned}&\text {Save} = \text {Prevent-Cost} - \text {Extra-Cost} \end{aligned}$$

**Table 7:** Summary of the vaccination cost per person without disabilities, the home vaccination cost per person with disabilities, the percentage of people with disabilities in our dataset, the successful identification rate of impedimented members, the calculated total extra cost for vaccination, and the calculated savings by eliminating vaccine impediments. We can achieve savings of more than $624 million by eliminating vaccine impediments.

Name	Abbreviation	Value
Vaccination cost per person without disabilities	$$\text {Cost}_{nd}$$	$80
Home vaccination cost per person with disabilities	$$\text {Cost}_{d}$$	$150
Percentage of people with disabilities in our dataset	$$\text {R}_d$$	0.25
Successful identification rate of impedimented members	$$\text {R}_s$$	0.614
Total extra cost for vaccination	Extra-Cost	$221,542,406
Total savings	Save	$624,408,749

## Conclusion

In this paper, we propose a flexible Bayesian method for predicting COVID-19 vaccination impediment scores under a Bayesian paradigm. Based on the accuracy of the results, we conclude that our proposed forecasting method performed better than the existing cutting-edge methods. The key findings of this study are:

- The proposed method, B-BMARS performed better than XGBoost, Gaussian Process, and Random Forest in terms of classification accuracy.
- Several important groups of variables are identified which could be the reasons for vaccine impediment, e.g., health risk and healthcare coverage.
- We identified four main categories of variables playing a key role in influencing the attitude of the public towards vaccines, including low household assets, high health risks, highly uninsured areas, and infrequent use of physician information.
- Interactions among some of these variables may play a crucial role in vaccine impediment, e.g. combining low medical coverage and low assets have more prediction power for vaccination impediment.
- We define the cause of COVID-19 vaccination impediments in these groups as due to physical barriers, psychological barriers, and health barriers. We then provide policy recommendations to reduce barriers from the perspective of each of these three barriers.
- Physical barriers can be explained by having less access to the vaccine, e.g., limited mobility, limited time, lack of transportation, and low vaccine supply. To overcome such barriers, we recommend that health care providers offer home care services, travel assistance, and arrange for special events, including vaccination camps.
- Psychological barriers refer to misconceptions or neglect of vaccines and can come from family, friends, social media, social norms, or good health. To overcome these barriers, we recommend that health care providers engage in community events and wellness activities, build stronger relationships and trust with insured members, and provide them with instructional videos or organize lectures that emphasize the health benefits and importance of vaccination.
- Health barriers are people’s existing health problems that make them more worried about the side effects of vaccines. To overcome such barriers, we recommend that health care providers obtain more information about people’s health, provide more specific advice to each individual, and use telemedicine to track their health after vaccination.
- We estimated the dollar benefit based on actual data and publicly available information resulting from our potential policy implications.

To the best of our knowledge, this is the first research that uses these flexible methods to analyze the data and arrive at conclusions that will have a significant impact on corporate decision making. Our findings have broad implications for solving complex problems with large datasets that require forecasting. Finally, our framework can have a direct impact on corporate and public policy related to future pandemics.

For future research, it is of great importance to expand the study to uninsured members and barriers in other countries. Equally important is how other types of data (e.g., image and text data), if available, can be incorporated to further improve predictive accuracy, better address vaccine barriers, and provide additional benefits. Additionally, we plan to enhance the scalability of the algorithm by employing parallel Markov Chain Monte Carlo (MCMC) within a simulated annealing framework^64^. This approach aims to enable the implementation of the algorithm in a single stage.

### Supplementary Information

Supplementary Information.

## Data Availability

The data files of the customer’s vaccine intentions and characteristics are available upon reasonable request from Prof. Mahajan (amahajan@mays.tamu.edu).
