# Supplementary material for: Identifying and overcoming COVID-19 vaccination impediments using Bayesian data mining techniques
Source: Sci Rep. 2024 Apr 13;14:8595. doi: 10.1038/s41598-024-58902-1 (PMC11016065; doi:10.1038/s41598-024-58902-1)
Supplement: Supplementary file 1 — Supplementary Information. [file 41598_2024_58902_MOESM1_ESM.pdf]

Supplementary Information

Identifying and overcoming COVID-19 vaccination impediments using Bayesian data mining techniques

Baseline Method: Extreme Gradient Boosting

Extreme gradient boosting (XGBoost)[1] is an implementation of an extended version of gradient boosting decision tree (GBDT)[2] which is an iterative algorithm based on decision trees and applicable to both regression and classification problems. Following the procedure and property of gradient boosting, XGBoost also belongs to ensemble learning where each decision tree works together and leads to a flexible model. On the one hand, it brings about powerful performance which makes it a key approach in applied machine learning and Kaggle competitions when dealing with structured or tabular data[3–5]. One of the main improvements is the normalization of the loss function reducing model variances and effectively avoiding overfitting. On the other hand, it greatly speeds up the execution and supports a variety of computing environments like parallelization and distributed computing.

Baseline Method: Gaussian Process

Gaussian process (GP) regression is also a popular method to determine the unknown function f[6,7]. Given variable $\mathbf{x}_i \in \mathbb{R}^p$ and response y_i $(i = 1, \cdots, n)$, the goal is to establish the pattern of f and predict the new y_* with input $\mathbf{x}_*$. The general formulation of a Gaussian process expresses the response y_i as a function of $\mathbf{x}_i$ with extra noise: $y_i = f(\mathbf{x}_i) + \varepsilon_i$, $\varepsilon_i \overset{\text{i.i.d}}{\sim} \mathcal{N}(0, \sigma^2)$. Additionally, it belongs to the Bayesian paradigm, which usually imposes a Gaussian prior on the unknown function f, and $\mathbf{f} = (f(\mathbf{x}_1), \cdots, f(\mathbf{x}_n))^\top$ follows a joint Gaussian distribution:

$$p(\mathbf{f}) \sim \mathcal{N}(\mathbf{f}|m(\mathbf{x}), \mathbf{K}), \quad [K_{ij}] = k(\mathbf{x}_i, \mathbf{x}_j), \tag{1}$$

where $m(\cdot)$ denotes the mean function and $k(\cdot, \cdot)$ refers to the covariance function.

More specifically, for the mean function $m(\mathbf{x})$, it reflects the expected function value at $\mathbf{x}$, i.e., $m(\mathbf{x}) = \mathbb{E}[f(\mathbf{x})]$, and a common choice is a constant mean function. When turning to the covariance function $k(\cdot, \cdot)$, it models the dependence between function values at different input points in a form of $k(\mathbf{x}_i, \mathbf{x}_j) = \mathbb{E}[(f(\mathbf{x}_i) - m(\mathbf{x}_i))(f(\mathbf{x}_j) - m(\mathbf{x}_j))]$. There are various choices, which are often decided according to the corresponding task[7]. Radial basis function (RBF) kernels are popular in capturing stationary and isotropic patterns. Another variant of RBF kernels has automatic correlation detection (ARD)[8] assigning different scale parameters to each variable instead of using a constant value[8], which can help select the key variables determining f. In addition, non-stationary kernels, such as the dot product kernel[9] and the more flexible deep network kernel[10] can also be a good choice in certain tasks. For the notations, we choose to use $\mathbf{D} = \{\mathbf{x}_{1:n}, \mathbf{y}_{1:n}\}$ to denote the input training data. When there is a new input $\mathbf{x}_*$, we can have a predictive distribution for the response y_*:

$$p(y_*|\mathbf{x}_*, \mathbf{D}) = \mathcal{N}(\mu_*, \sigma_*^2), \tag{2}$$

$$\mu_* = m(\mathbf{x}_*) + k(\mathbf{x}_*, \mathbf{x}_{1:n})(\mathbf{K} + \sigma^2\mathbf{I})^{-1}(\mathbf{y}_{1:n} - m(\mathbf{x}_{1:n})), \tag{3}$$

$$\sigma_*^2 = k(\mathbf{x}_*, \mathbf{x}_*) + \sigma^2 - k(\mathbf{x}_*, \mathbf{x}_{1:n})(\mathbf{K} + \sigma^2\mathbf{I})^{-1}k(\mathbf{x}_{1:n}, \mathbf{x}_*). \tag{4}$$

Non-parametric regression methods based on GP have received increasing attention and are widely used in various applications[11–13]. In addition, similar to BMARS, one solution to extend the model to classification tasks is to combine it with a probit model. However, when faced with relatively high-dimensional features, the model may be inaccurate if we do not have enough information about the predictive performance of each potential variable[7].

Baseline Method: Random Forest

Ensemble Learning[14] provides an alternative way of combining models, which is a popular procedure that constructs multiple weak learners and aggregates them into a stronger learner[15–17]. In several circumstances, it is challenging for an individual model to capture the unknown complex mechanism connecting inputs to the output(s) by itself. Therefore, it is a better strategy to use a divide-and-conquer method in the ensemble learning framework, which allows each of the models to fit a small part of the function. Ensemble learning's robust performance to handle complex data makes it a great candidate for many tasks.

Random forests (RF) is an ensemble learning model which can be used in classification, regression, and other tasks. RF usually constructs a large number of decision trees simultaneously during training time and lets them vote for the final predictions. More specifically, when faced with classification tasks, the predictions of RF, in the end, is the class selected by most trees. When turning to regression tasks, RF calculates the mean output of the individual decision trees as its prediction[18,19].

References

1. Chen, T. *et al.* Xgboost: extreme gradient boosting. *R package version 0.4-2* **1**, 1–4 (2015).
2. Natekin, A. & Knoll, A. Gradient boosting machines, a tutorial. *Front. neurorobotics* **7**, 21 (2013).
3. Naghibi, S. A., Hashemi, H., Berndtsson, R. & Lee, S. Application of extreme gradient boosting and parallel random forest algorithms for assessing groundwater spring potential using dem-derived factors. *J. Hydrol.* **589**, 125197 (2020).
4. Georganos, S. *et al.* Very high resolution object-based land use–land cover urban classification using extreme gradient boosting. *IEEE geoscience remote sensing letters* **15**, 607–611 (2018).
5. Chang, Y.-C., Chang, K.-H. & Wu, G.-J. Application of extreme gradient boosting trees in the construction of credit risk assessment models for financial institutions. *Appl. Soft Comput.* **73**, 914–920 (2018).
6. Schulz, E., Speekenbrink, M. & Krause, A. A tutorial on gaussian process regression: Modelling, exploring, and exploiting functions. *J. Math. Psychol.* **85**, 1–16 (2018).
7. Lei, B. *et al.* Bayesian optimization with adaptive surrogate models for automated experimental design. *Npj Comput. Mater.* **7**, 194 (2021).
8. Aye, S. A. & Heyns, P. An integrated gaussian process regression for prediction of remaining useful life of slow speed bearings based on acoustic emission. *Mech. Syst. Signal Process.* **84**, 485–498 (2017).
9. Williams, C. K. & Rasmussen, C. E. *Gaussian processes for machine learning*, vol. 2 (MIT press Cambridge, MA, 2006).
10. Wilson, A. G., Hu, Z., Salakhutdinov, R. & Xing, E. P. Deep kernel learning. In *Artificial intelligence and statistics*, 370–378 (PMLR, 2016).
11. Ceylan, Z. Estimation of municipal waste generation of turkey using socio-economic indicators by bayesian optimization tuned gaussian process regression. *Waste Manag. & Res.* **38**, 840–850 (2020).
12. Iyer, A. *et al.* Data-centric mixed-variable bayesian optimization for materials design. In *International Design Engineering Technical Conferences and Computers and Information in Engineering Conference*, vol. 59186, V02AT03A066 (American Society of Mechanical Engineers, 2019).
13. Snoek, J., Larochelle, H. & Adams, R. P. Practical bayesian optimization of machine learning algorithms. *arXiv preprint arXiv:1206.2944* (2012).
14. Sagi, O. & Rokach, L. Ensemble learning: A survey. *Wiley Interdiscip. Rev. Data Min. Knowl. Discov.* **8**, e1249 (2018).
15. Krawczyk, B., Minku, L. L., Gama, J., Stefanowski, J. & Woźniak, M. Ensemble learning for data stream analysis: A survey. *Inf. Fusion* **37**, 132–156 (2017).
16. Laradji, I. H., Alshayeb, M. & Ghouti, L. Software defect prediction using ensemble learning on selected features. *Inf. Softw. Technol.* **58**, 388–402 (2015).
17. Chen, X. M., Zahiri, M. & Zhang, S. Understanding ridesplitting behavior of on-demand ride services: An ensemble learning approach. *Transp. Res. Part C: Emerg. Technol.* **76**, 51–70 (2017).
18. Ho, T. K. Random decision forests. In *Proceedings of 3rd international conference on document analysis and recognition*, vol. 1, 278–282 (IEEE, 1995).
19. Denisko, D. & Hoffman, M. M. Classification and interaction in random forests. *Proc. Natl. Acad. Sci.* **115**, 1690–1692 (2018).
